# Supplementary material for: Frontline Science: Antagonism between regular and atypical Cxcr3 receptors regulates macrophage migration during infection and injury in zebrafish
Source: J Leukoc Biol. 2019 Sep 17;107(2):185–203. doi: 10.1002/JLB.2HI0119-006R (PMC7028096; doi:10.1002/JLB.2HI0119-006R)
Supplement: Supplementary file 4 — Supplementary Material [file JLB-107-185-s004.docx]

**Supplementary materials**

**Supplementary table I.** Top hits for predicted ligands of Cxcr3.2 and Cxcr3.3.

| Cxcr3.2 | Ligand name | C-score |
| --- | --- | --- |
| 1 | 0NN | 0.18 |
| 2 | DGW | 0.12 |
| 3 | 2CV | 0.5 |
| 4 | Y01 | 0.05 |
| Cxcr3.3 | Ligand name | C-score |
| 1 | DGW | 0.12 |
| 2 | 0NN | 0.10 |
| 3 | Y01 | 0.05 |
| 4 | 2CV | 0.04 |

**c-score-confidence index*

**Supplementary table II**. Accession numbers of sequences used fir the phylogenetic tree.

|  | Accession number |
| --- | --- |
| 1. Cavefish (CF) Ackr4 | ENST00000249887.2 |
| 1. Cavefish (CF) Ackr4b | ENSAMXG00000025769 |
| 1. Cavefish (CF)Cxcr3.2 | ENSAMXG00000035350 |
| 1. Cavefish (CF)Cxcr3.3 | ENSAMXG00000018866 |
| 1. Herring (CH) CXCR3 | XP_012694805.1 |
| 1. Cod (COD)Ackr3b | ENSGMOG00000019953 |
| 1. Cod (COD)Cxcr3.3 | ENSGMOG00000016951 |
| 1. Coelacanth (COE) Ackr2 | ENSLACG00000000319 |
| 1. Coelacanth (COE) Ackr3 | ENSLACG00000016030 |
| 1. Coelacanth (COE) Ackr4 | ENSLACG00000007877 |
| 1. Coelacanth (COE) Cxcr3.1 | XP_005999214.1 |
| 1. Coelacanth (COE) Cxcr3.2 | XP_014343707.1 |
| 1. Elephant shark (ES) Cxcr3 | XP_007909361.1 |
| 1. Frog (FR) Ackr3 | ENSXETG00000003296 |
| 1. Frog (FR) Cxcr3 | ENSXETG00000024989 |
| 1. Fugu (FU) Ackr4a | XP_003978861.2 |
| 1. Fugu (FU) Ackr4b | XP_011606262.1 |
| 1. Fugu (FU) Cxcr3.1 | XP_003966387.1 |
| 1. Fugu (FU) Cxcr3.3 | XP_003966388.2 |
| 1. Human (HU) Ackr1 | ENSG00000186810 |
| 1. Human (HU) Ackr2 | ENSG00000144648 |
| 1. Human (HU) Ackr3 | ENSG00000144476 |
| 1. Human (HU) Ackr4 | ENSG00000129048 |
| 1. Human (HU) Cxcr3 | ENSG00000186810 |
| 1. Lamprey (LAM) Ackr3 | XP_007909361.1 |
| 1. Mouse (MO) Ackr2 | ENSMUSG00000044534 |
| 1. Mouse (MO) Ackr3 | ENSMUSG00000044337 |
| 1. Mouse (MO) Ackr4 | ENSMUSG00000079355 |
| 1. Mouse (MO) Cxcr3 | ENSMUSG00000050232 |
| 1. Asian arowana (SF) Cxcr3 | XP_018587703.1 |
| 1. Asian arowana (SF) Cxcr3a | KPP61297.1 |
| 1. Spotted gar (SG) Ackr2 | ENSLOCG00000009409 |
| 1. Spotted gar (SG) Ackr4a | ENSLOCG00000018134 |
| 1. Spotted gar (SG) Ackr3 | ENSLOCG00000004865 |
| 1. Spotted gar (SG) Cxcr3.1 | XP_015224255.1 |
| 1. Spotted gar (SG) Cxcr3.3 | AWO96327.1 |
| 1. Zebrafish (ZF) Ackr3a | ENSDART00000090414 |
| 1. Zebrafish (ZF) Ackr3.2 | ENSDARG00000058179 |
| 1. Zebrafish (ZF) Ackr4a | ENSDART00000145446 |
| 1. Zebrafish (ZF) Ackr4b | ENSDART00000058703 |
| 1. Zebrafish (ZF) Cxcr3.1 | ENSDARG00000078177 |
| 1. Zebrafish (ZF) Cxcr3.2 | ENSDARG00000041041 |
| 1. Zebrafish (ZF) Cxcr3.3 | ENSDART00000146611 |
